# Improvement of chronic facial pain and facial dyskinesia with the help of botulinum toxin application

**DOI:** 10.1186/1746-160X-3-32

**Published:** 2007-08-22

**Authors:** Katharina Junghans, Saskia Rohrbach, Maik Ellies, Rainer Laskawi

**Affiliations:** 1 Department of Otorhinolaryngology, Head and Neck Surgery, University of Göttingen, Robert-Koch-Str. 40, D-37075 Göttingen, Germany

## Abstract

**Background:**

Facial pain syndromes can be very heterogeneous and need individual diagnosis and treatment. This report describes an interesting case of facial pain associated with eczema and an isolated dyskinesia of the lower facial muscles following dental surgery. Different aspects of the pain, spasms and the eczema will be discussed.

**Case presentation:**

In this patient, persistent intense pain arose in the lower part of her face following a dental operation. The patient also exhibited dyskinesia of her caudal mimic musculature that was triggered by specific movements. Several attempts at therapy had been unsuccessful. We performed local injections of botulinum toxin type A (BTX-A) into the affected region of the patient's face. Pain relief was immediate following each set of botulinum toxin injections. The follow up time amounts 62 weeks.

**Conclusion:**

Botulinum toxin type A (BTX-A) can be a safe and effective therapy for certain forms of facial pain syndromes.

## Background

The underlying mechanism of a chronic pain syndrome caused by alterations in the area of the trigeminal nerve seems to be an increased activity in trigeminal nerve fibers and an altered inhibition in the trigeminal nucleus. The increased neuronal activity (idiopathic or symptomatic) involves nociceptive neurons resulting in the perception of pain [1-3].

Various possible etiologies of chronic facial pain syndromes are known, including 1) disorders of the cranium, neck, eyes, ears, nose, sinuses, teeth, mouth and other facial structures and 2) cranial neuralgias, nerve trunk pain and deafferentiation pain [3]. Facial pain is often caused by cervical and other forms of dystonia, blepharospasm, hemifacial spasm, Meige-syndrome, masticatory hyperactivity, temporomandibular disorders (TMD), bruxism, trigeminal and other cranial neuralgias, tension-type headache or migraine.

Chronic facial pain can be difficult to manage [1]. One cause of the pain syndromes may be an affliction of the oral region in the form of lesions of peripheral trigeminal nerve fibers. Atypical facial pain is known to be initiated by surgical trauma in the oral region [4,5] and can also be induced by altered muscle function with hypertonicity [6].

There are numerous descriptions in the literature of patients with chronic facial pain or pain-associated dystonias effectively treated by injecting botulinum toxin into the involved areas [1,6-11], thus achieving total or partial relief of symptoms without the necessity of systemic medication with its often notable side effects. The long duration of the positive effects of botulinum toxin and the highly limited systemic complications associated with its use are important pharmacological features of this therapeutic option for the management of atypical facial pain and chronic pain syndromes.

It is difficult to explain the mechanisms leading to the analgesic effect of botulinum toxin used in the treatment of chronic facial pain or painful muscle disorders. Here we report an interesting case of facial pain with facial dyskinesia following dental surgery.

## Case presentation

A 53-year-old female patient who had been suffering for ten years from atypical facial pain combined with a partial facial spasm was referred to our outpatient clinic.

**Figure 1:**
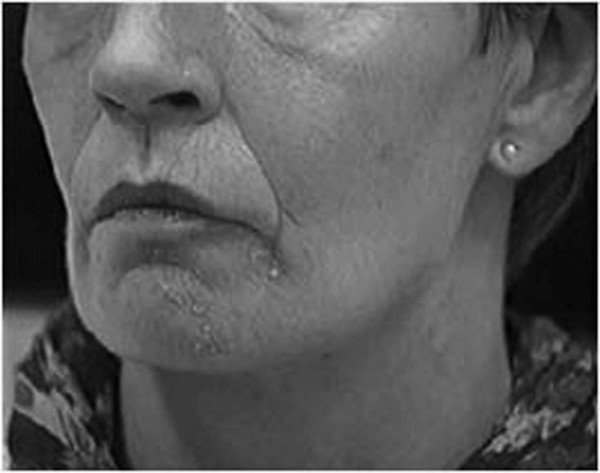
The eczema in the affected area disappeared after injection of BTX-A.

She presented with continuous distorsions of the mimic musculature in the region of the lower left lip, which had appeared following severe osteomyelitis of the left side of the mandible that had been treated surgically. For several weeks following the operation the patient experienced hypesthesia in the left mandibular region and skin. Thereafter, constant, disturbing spasms of the mimic musculature occurred combined with dyskinesia and deep spasmodic pain attacks located in her lower left lip region. In addition, a distinct cutaneous erythema appeared in the region of the dyskinesia (figure 1).

The patient reported that pain attacks occurred daily immediately after awakening in the morning, continued during the day without any improvement and subsided only at bedtime.

There had been no satisfactory response to various neurological or dental therapy attempts nor to acupuncture. Only therapy with carbamazepine had brought a slight and transient relief of her symptoms.

The patient felt herself immensely restricted by her symptoms and was socially and professionally disabled. She had had to retire because of the intolerable pain attacks, and reported having suicidal thoughts from time to time.

**Figure 2:**
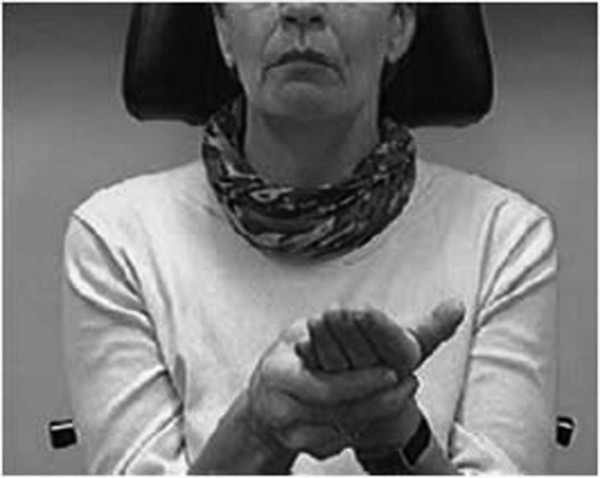
The figure shows the point in the left hand that the patient could press to stop the pain attacks and facial movements.

During the following years she detected alleviation points in her left hand and behind the left ear with which she was able to stop the convulsions and the pain as long as pressure was applied to the points (figure 2).

The patient had had no history of movement disorders such as hemifacial spasms nor of allergy, smoking or alcoholism. She had no history of medication except for carbamazepine.

On physical examination, no anatomic disorders, infections or tumors were found except for a discrete septum deviation. We observed continuous spasms in the region of her left lower lip, accompanied by an intense eczema in this region. She was able to stop the spasms and the pain by pressing the points on her hand or behind the ear.

After the patient had given informed consent, BTX-A-treatment was begun. She was treated over a period of 67 weeks with seven different injections of BTX-A at different time points.

The dose of BTX-A was increased from initially 5 units to 25 units at the seventh treatment. We also augmented the number of injection points from 2 points to 10 points in the affected area. Injections were made with 2.5 units per site (Botox^®^, Allergan Inc, Irvine, California; 0.1 ml = 2.5 units BTX-A). The time between the treatment sessions varied from 3 weeks to 24 weeks up to the last treatment. For details see table 1.

**Table 1:** Time course of treatment

Treatment	Dose of BTX-A administered	Number of injections (à 2.5 units)	Time of injection
1	5 units	2 points	Onset
2	10 units	4 points	after 5 weeks
3	15 units	6 points	after 2 weeks
4	20 units	8 points	after 3 weeks
5	20 units	8 points	after 11 weeks
6	25 units	10 points	after 17 weeks
7	25 units	10 points	after 24 weeks

Summary of treatment, dose, injection points and time points. BTX-A = botulinum toxin type A.

BTX-A was injected into the inferior depressor labii muscle in the left lower lip region. The seventh injection with 25 units injected into 10 points was the most effective with an effect lasting 24 weeks (table 1).

The patient was immediately pain-free after the injections and experienced other positive effects such as relief of spasms and eczema. The symptoms improved already after the first injection of botulinum toxin type A. At the check-up, three weeks after the first injection, the patient was free of symptoms and was very satisfied.

As agreed upon with the patient, she returned to our outpatient clinic for further treatment whenever any symptoms reappeared.

The BTX-A injection was repeated after 5 weeks with a total dose of 10 units at 4 injection points (4 injections à 2.5 units) because of mild spasms.

After the second injection, the patient again experienced a reduction in pain, spasms and eczema for a period of 7 weeks, at which time we injected 15 units into 6 injection points.

In the further course, the patient returned four more times after 3, 11, 17 and 24 weeks for further injections with 20 to 25 units BTX-A into 8 to 10 injection sites. Fourteen weeks after the last series, she reported in a telephone interview that the excellent positive effects were long lasting and that she was not suffering from pain, spasms or eczema.

The patient was able to reduce the dose of carbamazepine considerably.

In the course of the treatment period, the duration of the symptom-free period increased from a minimum of 3 weeks to 24 weeks. The longest positive effect was seen after the injection of 25 units BTX-A into 10 injection points in the lower left lip region.

The patient did not note any side effects except for a slight leakage at the corner of her mouth lasting a few days, which she did not find very irritating as the positive benefits were much more important for her. A total follow-up period of 62 weeks was observed in this patient.

In summary, the patient expressed great satisfaction and stated: "A completely new period in my life began" after the first injection.

## Discussion

Botulinum toxin has been used for 20 years to treat various neurological disorders associated with pathologically increased muscle tone or impaired autonomic nerve regulation [2,7,8,10-18]. In addition to the reduction in muscle innervation, botulinum toxin tends to reduce pain in focal dystonia, spasticity and other pain syndromes associated with muscle spasm [7,19-24]. An additional analgesic mechanism in muscle disorders associated with pain is conceivable, because pain relief does not necessarily correlate with the amount and duration of the neuromuscular effects [9,12].

Göbel et al. [15] reported several non-neuromuscular effects of the toxin as well as a normalization of increased muscle spindle activity, decompression of afferent nociceptive neurons of muscular and vascular tissue, retrograde intake of botulinum toxin in the peripheral and central nervous system with modulation of the central neuropeptide function, inhibition of sterile neurogenic inflammation and normalization of endplate dysfunction. It has been supposed that the alteration of the motor reflex activity may induce neuronal processes of central reorganization. BTX-A has also been shown to inhibit the release of substance P, a neurotransmitter responsible for activation of neurogenic inflammatory processes, from trigeminal nerve endings.

In our case, conclusive arguments pointing to the BTX A-effect responsible for the clear improvement of the patient's symptoms exist. One important point is the reproducible improvement following BTX-A application parallel to the reduced dyskinesia of the lower lip. Another point is the recurrence of extensive pain symptoms when the full BTX-A effect decreased. This is monitored and demonstrated by the simultaneous recurrence of pathological movements of the lower lip. Another argument is the statement of the patient that the pain symptoms disappeared completely after the BTX-A injections. However, it remains unclear whether the improvement is directly caused by a BTX-A effect or is only a secondary effect.

According to Göbel et al. [15], one can assume the existence of direct analgesic and neuromodulating mechanisms of botulinum toxin in the central nervous system, anti-inflammatory effects and effects on the myofascial tender points.

At the beginning of the 20^th ^century, Russel formulated the hypothesis of a trigemino-facial reflex positing that irritations of the trigeminal nerve lead to alterations in the facial motor nucleus, and that spontaneous activity of the facial nerve (muscle spasms) could occur. Dental, ophthalmic or otolaryngological diseases may act as trigger mechanisms in this connection [25]. On the other hand, the patient's complaints could be explained as a postsurgical pain syndrome due to chronic irritation of trigeminal nerve fibers following osteomyelitis and dental surgery. The simultaneous occurrence of pain and facial spasms might suggest that the rhythmic contractions of the facial muscles act as a facial trigger analogous to trigeminal neuralgia which can be caused by ectopic firing of injured nerve fibers [16].

According to Fromm et al. [2] and Göbel et al. [15], chronic alterations in the dental and oral region might induce degenerative changes in trigeminal axonal endings and a reduced inhibition in the trigeminal nucleus, leading to pain [6]. Their hypothesis was that disease of the trigeminal nerve causes increased firing as well as impaired the efficiency of the inhibitory mechanisms that control afferent activity in the trigeminal nucleus. The paroxysmal bursts of neuronal activity involve nociceptive trigemino-thalamic relay neurons and excruciating pain is experienced.

Another reason for the pain relief following botulinum toxin treatment is conceivable: painful perception may be secondary to the muscular spasm and caused by continuous contraction of the muscle fibers [26]. The painful muscle spasm is thought to be induced by regional muscle ischemia due to compression of blood vessels. An altered nociceptive processing is imaginable, leading to the perception of pain in the affected overactive muscles. It is assumed that continuous muscular contraction associated with dystonic muscular disorders induces a severe chronic pain syndrome [12]. Various disorders are often associated with painful sensations in the head and neck area [3,6-12] such as cervical dystonia, spasticity, hemifacial spasm, blepharospasm, temporomandibular joint syndrome or masseteric hypertrophy. The mechanisms of this phenomenon are poorly understood. The positive pharmacological effect could be thought to be achieved by various mechanisms: 1) blockage of cholinergic transmission and interruption of muscle contractions [7], 2) decompression of vascular nociceptive neurons, 3) normalization of muscle spindle activity (inhibition of γ motor endings [12]), or 4) modulation of central mechanisms with regard to neuropeptides and neurogenic inflammation [15].

BTX-A injections lead to a direct attenuation of these muscle contractions. An improvement in the aerobic muscular metabolism with regard to oxygen supply has also been postulated [6]. Cheshire [7] hypothesized that the beneficial effect in myofascial pain occurs through the interruption of muscle contraction by cholinergic denervation. According to Filippi [14], the most obvious mechanisms by which pain relief may be mediated are through a reduction of muscle spasm by cholinergic chemodenervation at motor end-plates and by inhibition of γ motor endings in muscle spindles [14].

A further point of interest is the correlation between the erythema in the affected area and the relief of this symptom by treatment with BTX-A. Borodic et al. [1] made the observation that the presence of erythematous patches or facial edema associated with severe pain has often been associated with the aggravation of pain. They discussed a non-neuromuscular effect of BTX-A which blocks edema, erythema, sensory discomfort and heat release and proposed the possibility of anti-inflammatory properties of botulinum toxin. Forty-four patients with chronic facial pain (diagnoses: TMD, headache, post-surgical pain syndromes, idiopathic trigeminal neuralgia) were treated with botulinum toxin injections. In 72% of their patients, they found erythematous discoloration or edema of the painful areas of skin which improved following BTX-A treatment. They noted that facial pains were frequently associated with varying degrees and manifestations of inflammatory responses and suggested this response to be a mechanistic component of pain. The presence of edema and erythema may be the outward physical signs of an inflammatory process [1]. The presence of cutaneous erythema suggests a pathogenesis involving inflammatory phenomena that have been well known to occur in myofascial pain syndrome, tension headache, temporomandibular disease, migraine, trigeminal neuralgia and post-surgical incisional pain syndromes.

Because the case described here showed a considerable correlation between muscle hyperactivity and pain perception, we hypothesize that facial pain may be induced 1) by mechanisms such as regional ischemia caused by vascular compression through continuous muscle contraction, 2) altered processing of nociceptive stimuli or 3) by irritation of trigeminal fibers following surgical trauma and contraction of neighboring muscle fibers. There is no clear explanation of why pressure applied to the retroauricular and hand "alleviation points" is able to interrupt the spasm and pain.

We conclude that injection of botulinum toxin type A is a safe, effective and long-lasting method that can be effective in certain cases of facial pain syndromes associated with muscular hyperactivity and inflammatory phenomena. It is important to mention that neither reproducible trials of the application of BTX-A for various forms of headache have been conducted to date nor has the mechanism of action for pain application been conclusively proven. For these reasons, the administration of BTX-A for chronic facial pain (without dyskinesia) should be reserved for those cases where conventional therapy proves ineffective and symptoms are severe. In addition, co-morbidity has to betaken into account. In such cases a multidisciplinary approach is needed [27].

## Competing interests

The author(s) declare that they have no competing interests.

## Authors' contributions

KJ performed clinical treatment, drafted the manuscript and participated in the literature research and revision of the manuscript. SR performed clinical treatment and participated in the revision of manuscript. ME performed clinical treatment and participated in the revision of the manuscript. RL perormed clinical treatment, participated in the literature research and revision of the manuscript and supervised the clinical treatment and scientific research. All authors read and approved the final manuscript.
